# Dental service utilisation and perceptions amongst Indian rural children with intellectual and developmental disabilities

**DOI:** 10.1007/s40368-026-01175-1

**Published:** 2026-02-12

**Authors:** Philcy Philip, Mathew lim, Gregory Armstrong, Nathan Grills

**Affiliations:** 1 Nossal Institute for Global Health, University of Melbourne, Melbourne, Australia; 2 Bangalore Baptist Hospital, Bengaluru, India; 3 Melbourne Dental School, University of Melbourne, Melbourne, Australia; 4 Global & Cultural Mental Health Unit, University of Melbourne, Melbourne, Australia; 5 Nossal Institute for Global Health, University of Melbourne, Melbourne, Australia; 6 Australia India Institute, University of Melbourne, Melbourne, Australia

**Keywords:** Dental visits, Perceived need, Children with intellectual and developmental disability, Rural, India

## Abstract

**Purpose:**

The present study investigated the factors affecting dental visits and the perceived need for dental care amongst children and adolescents with intellectual and developmental disabilities (IDD) living in rural India.

**Methods:**

A cross-sectional study was conducted using a convenience sample of 160 caregivers, of whom 79% were mothers with a mean age of 32 ± 8.67 years, caring for children with a mean age of 7.45 ± 3.72 years. The caregivers completed a questionnaire that explored various factors affecting dental service utilisation and perceived need for dental care. Indicators of influencing factors were adopted from validated instruments assessing healthcare access and including demographic and socioeconomic variables. Simple univariable logistic regression models were fitted to determine the odds of dental visits and perceived dental need.

**Results:**

Twenty-three per cent of participants reported dental visits, whilst 29% perceived a need for dental care. Factors that significantly affected dental visits were demographic variables such as type of disability (OR 3.34; 95% CI 1.32, 8.48), child’s age (OR 4.87; 95% CI 1.59, 14.91), and the availability of information regarding oral care (OR 3.51; 95% CI 1.56, 7.90). Other factors that significantly affected dental utilisation were caregivers’ perception regarding child’s oral health (OR 3.67; 95% CI 1.68, 8.02) and the proximity to dental clinics (OR 4.18; 95% CI 1.56, 11.15). Similarly, these variables were associated with increased odds of perceived need for dental care, except for the caregiver’s perception of the child’s oral health (OR 0.21; 95% CI 0.09, 0.46). Additionally, caregivers' perception of the quality of dental care impacted their perceived need for care (OR 0.79; 95% CI 0.67, 0.94).

**Conclusion:**

Dental visits and perceived need for care were low amongst children and adolescents with IDD in rural India. The lack of information, proximity to dental clinics and quality of care affected dental service utilisation.

**Supplementary Information:**

The online version contains supplementary material available at 10.1007/s40368-026-01175-1.

## Introduction

The Diagnostic and Statistical Manual of Mental Disorders, Fifth Edition, defines intellectual and developmental disability (IDD) as a neurodevelopmental disorder that begins during the developmental period, which affects cognitive functioning and adaptive behaviour (APA 2013). Adaptive impairments are noted when the child fails to meet social and cultural standards, whilst standardised intelligence tests confirm cognitive limitations. In addition, symptoms such as learning delay and poor problem-solving skills confirm the diagnosis (Girimaji et al. 2020). However, diagnostic criteria may vary internationally; for example, India’s National Sample Survey defines IDD in children under 18 as lacking comprehension, communication, daily living skills, and problem-solving ability (National Sample Survey 2003).

Children with IDD have a higher prevalence of oral diseases than those without IDD (Anders and Davis 2010; Ward et al. 2019; Wilson et al. 2019; Zhou et al. 2017). A main reason is that cognitive and adaptive impairment can affect oral health, as neurodevelopmental deficits can limit physical abilities and manual dexterity, making oral care, such as using a toothbrush, difficult, whilst cognitive impairments reduce their ability to comply with oral hygiene instructions (Ziegler and Spivack 2018). This leads to poor oral hygiene, increasing the risk of oral diseases. Additional factors such as parafunctional habits, altered dental morphology, and multiple medications can also affect oral health, especially by reducing saliva production and its protective functions (Cooper et al. 2015; Xavier et al. 2013). Overall, children and adolescents with IDD exhibit an increased susceptibility to oral diseases compared to their neurotypical peers, primarily due to inherent functional and cognitive impairment.

In addition, poor oral health amongst children with IDD can be attributed to the lack of universal access to dental care, which is affected by individual, organisational, and system-level barriers (Da Rosa et al. 2020). Lack of bodily awareness, altered pain response, limited communication abilities, or atypical expressions may affect an individual with IDD’s ability to convey oral issues (Gilbert-MacLeod et al. 2000). As a result, oral health issues may go unrecognised or misinterpreted (Alborz et al. 2005). Organisational and system-based challenges, such as limited service availability and limited practitioner willingness or preparedness to treat individuals with IDD, continue to impede access to care (Lim et al. 2021; Ningrum et al. 2021). These challenges are compounded by a global shortage of training programmes in special care dentistry, further limiting service provision for this population.

In 2016, the Indian government ratified the Right to Persons with Disability Act, which guarantees equal and barrier-free access to health care for people with disabilities (Department of Empowerment of Persons with Disabilities 2016). However, many studies conducted in India (Mehta et al. 2024; Philip et al. 2024b; Philip et al. 2024c) have reported poorer oral health and fewer dental visits amongst children with IDD than their peers (Ningrum et al. 2021; Philip et al. 2024a). These findings underscore a disparity in access to dental care in India. With 1–3% of India's population having an intellectual disability (Russell et al. 2022), and with their increased susceptibility to poor oral health, the result is a substantial oral health burden on individuals and communities (Mehta et al. 2024).

Exploring dental service utilisation in rural India is imperative as a considerable proportion of people with disabilities reside there (Rashmi and Mohanty 2024). Remoteness has been shown to negatively affect dental visits and oral health outcomes (Sawhney et al. 2023); however, most dental surveys amongst individuals with IDD in India have focussed on urban residents (Philip et al. 2024a). Although the differences in distribution of individuals with IDD between urban and rural areas are not stark, rural areas lack adequate facilities, identification mechanisms, and referral pathways (Lakhan et al. 2015). Dental awareness has been traditionally low in these areas (Kapoor et al. 2023). Fewer dentists practice in rural areas than in urban areas, and most rural dentists are non-specialists, restricting the scope of services for individuals with IDD (Tandon 2004). Therefore, access is likely to be further restricted in rural areas, increasing the risk of dental diseases.

Understanding the factors affecting access to dental care for children with IDD in rural areas is essential to developing interventions to improve care. Thus, the present study aimed to investigate the determinants and patterns of dental care utilisation and perceived dental needs amongst individuals with IDD aged 2–19 years living in rural India.

## Methods

### Study area and context

Bengaluru is the capital city of the Karnataka state, located in the south of India with a population of 18 million. Bengaluru is divided into Bengaluru urban and Bengaluru rural based on population density and economic activity. Rural Bengaluru is less densely populated (431/km^2^) than the urban Bengaluru (4381/km^2^), with much of the population involved in agricultural activities. Four subdistricts (taluks) form rural Bengaluru with a total size of 2298 sq. km (*Bangalore Rural District*, 2025). The Government of Karnataka initiated District Disability Rehabilitation Centres (DDRCs) in rural Bengaluru consistent with requirements under the Rights for Persons with Disabilities Act 2016 (DDRC Scheme: Department of Empowerment of Persons with Disabilities, 2019); DDRCs focus on rural or semi-urban populations, providing identification, early intervention, and therapeutic services for individuals with IDD.

### Study design and data collection

A cross-sectional survey was conducted amongst caregivers of children with IDD attending the DDRCs in various rural suburbs of Bengaluru whose diagnosis had been confirmed by the in-house psychologist. Convenience sampling for data collection was used, as a comprehensive list of children with IDD was unavailable from which to obtain a probability-based sample of carers. Paper-based questionnaires were distributed to those who consented to our invitation to participate; DDRC staff, field workers, and the primary investigator distributed the questionnaire. All questions were in Kannada and English to accommodate caregivers’ language preferences and ensure clarity in responses. Assistance was provided by the DDRC staff for those with reading or writing difficulties. Questionnaires were distributed at the DDRCs, DDRC follow-up camps, and to families of children with IDD during home visits. Data collection occurred for 4 months starting from August of 2023. A total of 160 caregivers of children and adolescents with IDD responded to our invitation.

### Eligibility criteria

- The caregiver should be registered with the DDRC.
- The child or adolescent in caregiver’s care should be diagnosed with any of these conditions: intellectual disability (ID), autism spectrum disorder (ASD), cerebral palsy (CP), or other neurodevelopmental disorders (global developmental delay, speech and language delay, or attention-deficit/hyperactivity disorder and Down syndrome) (National Sample Survey 2003).
- The child or adolescent in the caregiver’s care should be between the ages of 2 and 19 years.
- The caregiver should willingly provide consent and be cognitively able to answer questions.

Ethics approval was obtained from the Human Research Ethics Committee, the University of Melbourne (2023-25495-37,142-4) and the Bangalore Baptist Hospital (BBH/IRB/2023/002).

### Measuring tool and framework used

Validated questions were selected from published questionnaires (Cu et al. 2021; Haggerty and Levesque 2015; Lakshmi et al. 2019; Nelson et al. 2011; Waterworth et al. 2022). The questionnaire used was developed based on protocols recommended by De Vaus (2014), which involved three steps: clarification of concepts, identifying pre-existing indicators, and evaluating indicators. The questionnaire was pretested amongst 10 caregivers and reviewed by authors (NG, GA, and ML) before distribution. This resulted in rewording/modification or exclusion of some of the questions and restructuring the order of questions. Although the questionnaire was revised to improve clarity and relevance, the updated version was not retested due to logistical and resource constraints.

### Variables measured

The questionnaire was used to collect data on behaviour relating to dental visits and various attributes influencing those visits. It consisted of three sections, investigating (1) demographic variables, (2) number of dental visits, and (3) perceived need. Each section had follow-up questions to explore reasons and features. A dental visit was defined as any time the caregiver took their child to a dental clinic within the past 12 months. The perceived need for a dental visit was determined by whether the caregiver felt their child required dental care but could not avail themselves of care.

The follow-up questions (16 close ended with Yes/No options or multiple choice) for those who visited focussed on attributes that influenced dental visits, such as reasons for seeking dental care, the frequency of visits, satisfaction with the services received, types of dental clinics visited, challenges in reaching the clinic, time taken to get there, the child’s cooperation, waiting times, attitudes of the dentists and staff, facilities available at the clinic, comprehension of the information provided by the dentists, and whether the dentists arranged follow-up appointments.

Additionally, the questionnaire explored the reasons for not visiting a dentist and perceived needs through questions that offered multiple-choice answers alongside an open-ended option. General inquiries examined knowledge and attitudes, including the caregiver’s confidence in identifying dental issues in their child, their perspective on preventive oral care, information received about oral health, and the number of clinics available in their vicinity. These aspects were measured using ordinal and nominal response types.

Two questions addressed caregivers’ impressions of the quality of dental care and the difficulty of accessing a dental clinic, using an interval-level response scale from one to ten, with ten indicating the greatest difficulty and best quality. The questionnaire included information regarding the type of disability and its severity, which was subsequently reconfirmed by the in-house psychologist.

The questionnaire also examined parents’ perceptions of their child’s oral health, serving as a proxy for normative need. Parents who rated their child’s dental health as average to very poor were categorized as having high needs, whereas those who rated it as good to excellent were classified as having low needs.

### Statistical analysis

The initial analysis was descriptive to describe dependent and independent variables, followed by bivariate analysis to describe the prevalence of dental visits and perceived need across various demographics and covariates. Follow-up questions were treated as independent variables and expressed and perceived need as dependent variables. Simple logistic regression models were fitted to describe the unadjusted odds ratio, p values, and 95% confidence intervals of the various independent variables on expressed and perceived need. STATA 18, (StataCorp. 2023) was used for the statistical analysis.

## Results

### Description of participants

One hundred and sixty caregivers of children and adolescents with IDD responded to the invitation; three caregivers did not meet the inclusion criteria. Most caregivers were parents or a close relative; 80% were female, with a mean age of 32.15 ± 8.67 years, and 72% had completed secondary education (Table 1). Fifty-six per cent of children were male, with a mean age of 7.45 ± 3.72 years. Thirty-eight per cent had intellectual disabilities (ID) as their primary diagnosis, whilst 30% had other neurodevelopmental disorders, and 24% had CP. The severity of the disability was mild in 47% and severe in 17% of children. The majority resided in various taluks of rural Bengaluru. Sixty per cent felt their child’s dental care needs were low.

**Table 1:** Participant and child demographic, self-assessed dentition status, attitude towards dental care, information received regarding child’s oral care, distribution of clinics and self-perception regarding the quality of dental care

Independent variables	*n* (%)
Gender of carer*	
Male	31 (19.8)
Female	125 (79.6)
Childs gender*	
Male	84 (56.3)
Female	65 (43.6)
Childs age*	
1–5yrs	48 (30.7)
6–11yrs	88 (56.4)
12–19yrs	20 (12.8)
Type of disability*	
ID with and without other disorders	57 (37.5)
ASD with and without other disorders	14 (9.2)
CP with and without other disorders	36 (23.7)
Other neurodevelopmental disorders **(**ADHD, GDD, SLD, DS)	45 (29.6)
Severity of disability*	
Mild	55 (47.4)
Moderate	41 (35.3)
Severe	20 (17.2)
Taluk (Subdistrict)*	
Surrounding taluks	32 (21.0)
Bengaluru rural	120 (78.9)
Caregivers education*	
< Primary education	38 (25.1)
Primary and secondary education	92 (61)
> Secondary Education	21 (13.9)
Breadwinners’ occupation*	
Unskilled & unemployed	30 (20.2)
Clerical, shop-owner/farmer	35 (23.6)
Semi-skilled	36 (24.3)
Semi-professional & skilled	47 (31.7)
Perceived child’s dental health*	
High need	62 (39.7)
Low need	94 (60.3)
Do you feel confident you can identify dental issues in your child?*	
Always	85 (55.6)
Most of the time	38 (24.8
Sometimes to Never	30 (19.6)
How much do you agree that dental disease can be prevented?*	
Strongly agree	35 (23.5)
Agree	105 (70.5)
Disagree to don’t know	9 (6.0)
Have you received any information regarding caring for your child’s teeth?*	
No	85 (55.9)
Yes	67 (44.1)
What best describes the number of clinics in your neighbourhood?*	
Few clinics around	30 (21.1)
Some to many clinics around	23 (16.2)
None for many km	89 (62.7)
	Mean (SD)
How much would you rate the overall quality of dental care in your community (1 = very poor, 10 very good)	7.95 (± 2.13)

^*^Total number of participants is short of 157 due to missing data

A majority of caregivers (55.6%) felt confident in identifying dental needs in their child’s teeth. More than half had received no information regarding dental care, and 63% mentioned very few nearby clinics.

### Prevalence of dental visits and perceived needs and related findings

The prevalence of dental visits was 23% in the last 12 months, whilst 29% needed dental care but could not visit (Table 2). Among those who utilised dental services, 81% were either for consultation or screening (Appendix 1), 45% visited only in an emergency, and the rest more regularly, i.e. 27% once a year and 18.8% once in 6 months. Travelling to the nearest clinic took more than 30 min in 65% of those with dental visits. The mean score of ease of access to a clinic score was 5.42 ± 3.3 out of 10. Most reported being satisfied with care (88%) and had minimal barriers to accessing dental care (supplementary table). The top reason for not visiting a dental clinic was the lack of need for dental care (50.4%) (Fig. 1 Appendix). The top three reasons reported by those who perceived the need for dental care but could not were distance from the clinic (31.7%), lack of time (22%), unawareness of what to do (19.5%) and cost (17.1%) (Fig. 2 Appendix).

**Table 2:** Distribution of outcome variables: dental visits and perceived need (*n*%)

N 157	*n* (%)
Dental visit in past 12 months (expressed need)	
No	121 (77.1)
Yes	36 (22.9)
Perceived the need to visit a dentist but could not in the past 12 months (perceived need) *	
No	103 (71.5)
Yes	41 (28.5)

^*^Total number of participants is short of 157 due to missing data

### Factors affecting dental visits

In unadjusted regression analyses, there were higher odds of dental visits in the 6–11-year-olds (OR 4.87; 95% CI 1.59, 14.91) compared to 1–5-year-olds (Table 3). Similarly, there were relatively higher odds of dental visits amongst children with CP (OR 3.34; 95% CI 1.32, 8.48) than those with ID. Odds of dental visits were higher amongst those who perceived poor oral health in their child (OR 3.67; 95% CI 1.68, 8.02) than those with good oral health. The availability of information regarding dental care (OR 3.51; 95% CI 1.56, 7.90) and the number of clinics in the vicinity improved the odds of dental visits (OR 4.18; 95% CI 1.56, 11.15). Time taken for travel and distance were issues, and most respondents expressed moderate difficulty getting to the dental clinic.

**Table 3:** Prevalence of dental visit based on independent variables with unadjusted odds ratio and 95% Confidence interval

Independent variables	Dental visits, *n* (%)	Crude OR	
		OR (95%CI)	*p* value
Gender of carer			
Male	7 (22.6)	1.00	–
Female	28 (22.4)	0.99 (0.39,2.54)	0.983
Childs gender			
Male	17 (20.2)	1.00	–
Female	17 (26.2)	1.39 (0.65,3.00)	0.395
Childs age			
1–5 years	4 (8.3)	1.00	
6–11 years	27 (30.7)	4.87 (1.59,14.91)	**0.006**
12–19 years	5 (25)	3.60 (0.86,15.47)	0.077
Type of disability			
ID with and without other disorders	11 (19.3)	1.00	–
ASD with and without other disorders	1 (7.1)	0.32 (0.04,2.7)	0.298
CP with and without other disorders	16 (44.4)	3.34 (1.32,8.48)	**0.011**
Other neurodevelopmental disorders (ADHD, GDD, SLD, DS)	6 (13.3)	0.64 (0.22,1.9)	0.424
Severity of disability			
Mild	10 (18.2)	1.00	
Moderate	5 (12.2)	0.62 (0.19,1.99)	0.427
Severe	5 (25)	1.50 (0.44,5.09)	0.516
Taluk (Subdistrict)			
Surrounding taluks	6 (18.8)	1.00	
Bengaluru rural	29 (24.2)	1.38 (0.52,3.68)	0.519
Caregivers education			
< Primary education	11 (28.9)	1.00	
Primary and secondary education	15 (16.3)	0.48 (0.19,1.16)	0.105
> Secondary education	9 (42.9)	1.84 (0.60,5.60)	0.282
Breadwinners’ occupation			
Semi-professional and skilled	7 (14.9)	1.00	
Unskilled and unemployed	7 (23.3)	1.7 (0.54,5.58)	0.352
Clerical, shop-owner/farmer	6 (17.1)	1.18 (0.36,3.88)	0.783
Semi-skilled	11 (30.6)	2.51 (0.86,7.34)	0.092
Perceived child’s dental health			
Low need	13 (13.8)	1.00	
High need	23 (37.1)	3.67 (1.68,8.02)	**0.001**
Confident identifying dental issues			
Always	21 (24.7)	1.00	
Most of the time	8 (21.1)	0.81 (0.32,2.04)	0.660
Sometimes to never	6 (20)	0.76 (0.27,2.11)	0.602
Dental disease can be prevented			
Strongly agree	8 (22.9)	1.00	
Agree	23 (21.9)	1.05 (0.42,2.64)	0.906
Disagree to Don’t know	4 (44.4)	0.37 (0.08,1.71)	0.204
Received information regarding oral care			
No	11 (12.9)	1.00	
Yes	23 (34.3)	3.51 (1.56,7.90)	**0.002**
Number of clinics in your neighbourhood			
None for many km	16 (17.9)	1.00	
Few clinics around	5 (16.7)	0.91 (0.30,2.74)	0.871
Some to many clinics around	11 (47.8)	4.18 (1.56,11.15)	**0.004**
Rating of overall quality of dental care	Mean (SD)		
	7.96 (± 2.17)	1.00 (0.84,1.19)	0.960

^*^p < 0.05 significant

### Factors affecting perceived dental need

In unadjusted regression analyses, factors that affected whether carers perceived a need for dental care but could not visit were the child’s age, type of disability, the prevalence of dental diseases, the availability of information regarding dental care, and caregivers’ impression of the quality of dental care (Table 4). Carers of children with disabilities aged 6–11 had higher odds (OR 5.12; 95% CI 1.82, 14.41) of perceiving the need for dental care than children aged 1–5. Similarly, children with CP (OR 2.66; 95% CI 1.07, 6.61) had increased odds of perceived need compared to those with ID. The odds of perceived need were lower amongst those with higher dental needs in their child than those with low need (OR 0.2; 95% CI 0.09, 0.46). In contrast, those who received information regarding dental care had higher odds of perceived need (OR 3.31; 95% CI 1.55, 7.06) than those who did not receive information regarding dental care. Caregiver impression regarding the quality of dental care affected perceived need; there was a 0.79 (95% CI 0.67, 0.94) decrease in odds of perceived need with every one-degree increase in score regarding the quality of dental care.

**Table 4:** Prevalence of perceived need for dental visits based on independent variables with unadjusted odds ratio and 95% Confidence interval

Independent variables	Perceived need, *n* (%)	Crude OR		
		OR (95%CI)		*p* value
**Gender of Carer**				
Male	9 (30)	1.00		
Female	31 (27.4)	0.88 (0.36,2.13)		0.781
**Childs gender**				
Male	22 (27.5)	1.00		
Female	17 (27.4)	0.99 (0.23,0.62)		0.991
**Childs age**				
1–5 years	5 (10.9)	1.00		
6–11 years	30 (38.5)	5.12 (1.82,14.41)		**0.002**
12–19 years	6 (30.0)	3.51 (0.92,13.32)		0.065
**Type of disability**				
ID with and without other disorders	13 (24.1)	1.00		
ASD with and without other disorders	2 (18.2)	0.70 (0.13,3.67)		0.674
CP with and without other disorders	16 (45.7)	2.66 (1.07,6.61)		0.036
Other neurodevelopmental disorders (ADHD, GDD, SLD, DS)	10 (25)	1.05 (0.41,2.72)		0.918
**Severity of disability**				
Mild	9 (17)	1.00		
Moderate	11 (28.2)	1.92 (0.71,5.22)		0.201
Severe	7 (36.8)	2.85 (0.88,9.24)		0.081
**Taluk (Subdistrict)**				
Surrounding taluks	10 (34.4)	1.00		
Bengaluru Rural	29 (25.9)	0.66 (0.27,1.59)		0.359
**Caregivers’ Education**				
< Primary education	8 (21.6)	1.00		
Primary and secondary education	23 (27.7)	1.38 (0.55,3.48)		0.483
> Secondary education	8 (44.4)	2.9 (0.86,9.77)		0.086
**Breadwinners’ occupation**				
Semi-professional and skilled	16 (35.6)	1.00		
Unskilled & unemployed	9 (31.0)	0.82 (0.30,2.21)		0.688
Clerical, shop-owner/farmer	6 (19.4)	0.44 (0.15,1.28)		0.131
Semi-skilled	6 (19.4)	0.44 (0.15,1.28)		1.131
Perceived child’s dental health				
Low need	28 (46.7)	1.00		
High need	13 (15.7)	0.21 (0.09,0.46)		** < 0.001**
Confident identifying dental issues				
Always	26 (32.9)	1.00		
Most of the time	8 (22.9)	0.60 (0.24,1.51)	0.282	
Sometimes to never	7 (24.1)	0.65 (0.25,1.71)	0.382	
Dental disease can be prevented				
Strongly agree	12 (37.5)	1.00		
Agree	24 (24.7)	0.54 (0.23,1.28)	0.166	
Disagree to Don’t know	3 (33.3)	0.83 (0.17,3.96)	0.819	
Received information regarding oral care				
No	15 (18.3)	1.00		
Yes	26 (42.6)	3.31 (1.55,7.06)	**0.002**	
Number of clinics in your neighbourhood				
None for many km	10 (35.7)	1.00		
Few clinics around	8 (40)	1.2 (0.37,3.91)	0.76	
Some to many clinics around	21 (24.4)	0.58 (0.25,1.20)	0.25	
Rating of overall quality of dental care	Mean (SD)			
	7.10 (± 2.23)	0.79 (0.67, 0.94)	**0.007**	

^*^p < 0.05 significant

## Discussion

The present study highlights that expressed needs (i.e. actual dental visits in the past 12 months) and unexpressed needs (i.e. a perceived need for a dental visit) were low amongst caregivers of children and adolescents with IDD in rural Bengaluru. Factors that affected dental visits included the child's age, type of disability, caregivers’ perception of their child’s dental need, and the availability of information on oral care. In addition, the distribution of clinics in the vicinity affected dental visits. Unexpressed or perceived needs were also affected by the age of the child, type of disability, need for dental care, and availability of information regarding care. Other covariates that affected unexpressed need included the caregivers' impressions of the quality of dental care in the community. Most dental visits were need-based.

The prevalence of dental visits for IDD was 23%, substantially lower than reported previously in urban Bengaluru amongst children with DS (57%) (Sabbarwal et al. 2018) and children with ASD (53%) (Richa et al. 2014). The difference indicates a disparity in access in rural areas, which may be due to the disproportionately low distribution of dentists in rural areas, as the relative odds of dental visits were higher in areas with a good distribution of dentists. And less than half (48%) of the participants lived in areas with good dentist distribution. The finding concurs with other reports regarding the low distribution of dentists in rural India (Tandon 2004), and probably indicates a correlation between dental care demand and the density of dental practices in the vicinity. Sabbarwal and colleagues reported that, amongst children with DS, the odds of dental visits increased with improved accessibility. Thus, enabling more dentists to practice in rural areas may improve dental attendance (ref).

Only a quarter of caregivers visited a dentist or reported a perceived need for dental care for their child, indicating a low demand for dental care. Whilst previous research has shown that region, parental education, family structure, and household income influence dental attendance (Sawhney et al. 2023), none of these variables affected dental visits or perceived need in this cohort. Instead, the availability of dental care information was an important determinant of both dental visits and felt need. Similar findings were reported amongst caregivers of children with DS in Bengaluru, where those who received oral health information were more likely to utilise oral health services (Sabbarwal et al. 2018). Providing caregivers with information about preventive oral care has been shown to improve visit routines and reduce problem-based dental visits (Camargo et al. 2012).

Despite the importance of accessible information, we were unable to identify any printed or online material regarding dental care, published in India or in local languages that specifically supports caregivers of children with intellectual disabilities. This aligns with previous reports highlighting the limited availability of information on oral care for caregivers (Ummer-Christian et al. 2018). Consequently, opportunities to enhance awareness—and thereby improve dental attendance—remain restricted in the Indian context. Improving caregivers’ knowledge of preventive dental care through improved access to culturally and linguistically appropriate information, alongside establishing regular dental screenings at dental clinics, DDRCs, and other early intervention centres, may facilitate early identification of oral diseases and promote timely care.

Caregivers’ perception of dental care quality significantly influenced perceived need. There are very few quality checks or standards for dental care for those with IDD in India. The NABH and the DCI have recommendations (NABH 2023; DCI 2017), but they are not mandatory. They recommend providing wheelchair access to clinics; however, most rural clinics cannot meet these requirements, as clinic spaces are rented and may not be disability inclusive. Enabling dentists to facilitate disability care and regular quality audits could improve the perceived need for care.

A strength of the present study is the use of composite indicators: frequency of visits, reasons for visits, and whether the patient visited a regular dental practitioner (Lopez Silva et al. 2021). Most studies exploring access to dental care focus on service utilisation but overlook the reasons behind limited utilisation. In addition, very few studies have examined perceived need amongst children with IDD in rural India (Philip et al. 2025), and our study is the first published study to explore this parameter in India. However, limitations include the sample size and non-probability sampling, which may affect generalisability. Furthermore, given that DDRCs were the most effective way of recruiting, we may have under-sampled those with severe disabilities or those who may have had difficulty accessing DDRCs. Secondly, oral health needs were self-evaluated rather than validated by the clinician, and such approaches tend to underestimate dental needs (Hennequin et al. 2000). A final drawback is the present study’s inability to account for confounders due to the small sample size; hence, findings are unadjusted estimates.

## Conclusion

Our findings highlight factors that affect dental visits and the perceived need for dental care amongst children and adolescents with IDD in rural India. Limited access to oral health information, along with concerns about the quality of available services, affected their perceived need for care. Meanwhile, the availability of clinics in the vicinity and the type of disability strongly affected dental visits. Nevertheless, both dental attendance and the perceived need for dental care need to improve, underscoring the need for targeted strategies to strengthen service utilisation. Improving caregivers’ oral-health knowledge, expanding the availability of disability-inclusive dental services, and establishing minimum quality standards for dental clinics may enhance access to timely and appropriate care. Such measures are essential for reducing oral-health disparities and supporting better outcomes for children with IDD in rural Bengaluru.

## Supplementary Information

Below is the link to the electronic supplementary material.

Supplementary file1 (DOCX 56 KB)

## Data Availability

No datasets were generated or analysed during the current study.
