# Supplementary material for: Dental service utilisation and perceptions amongst Indian rural children with intellectual and developmental disabilities
Source: Eur Arch Paediatr Dent. 2026 Feb 12;27(1):229–39. doi: 10.1007/s40368-026-01175-1 (PMC12963194; doi:10.1007/s40368-026-01175-1)
Supplement: Supplementary file 1 — Supplementary file1 (DOCX 56 KB) [file 40368_2026_1175_MOESM1_ESM.docx]

**Supplementary data**

**Supplementary Table 1:** Describing pattern of dental visits with possible barriers e.g. child’s cooperation, waiting time, staff attitude, adequacy of facilities, satisfaction with care, adequacy of information provided and difficulty getting to the dentists

| N | n(%) | |
| --- | --- | --- |
| Reason for visit *( n=32)* |  |  |
| Consultation | 18(56.2) | |
| Screening | 8(25.0) | |
| Treatment | 6(18.8) | |
| Is there one dental clinic/dentist you regularly visit for your child’s dental care *( n=33)* | | |
| No | 20(60.6) | |
| Yes | 13(39.4) | |
| How often do you visit your dentists ( *n=33)* |  |  |
| No regular pattern | 3(9.1) | |
| Once a year | 9(27.3) | |
| Once every six months | 6(18.2) | |
| Only in need | 15(45.5) | |
| Type of clinic visited *(dental visit n=33)* |  |  |
| DDRC | 23(69.7) | |
| Dental College | 1(3.0) | |
| Private Dental Clinic | 9(27.3) | |
| Travel time>30min *(n=31)* |  |  |
| No | 11(35.5) | |
| Yes | 20(64.5) | |
| Was the child cooperative during treatment/ consultation *(n=32)* |  |  |
| No | 6(18.8) | |
| Yes | 26(81.3) | |
| Long waiting time at the clinic *(n=22)* |  |  |
| No | 14(63.6) | |
| Yes | 8(36.4) | |
| Were the dental staff supportive *(n=31)* |  |  |
| Supportive | 30(96.8) | |
| Unsupportive | 1(3.2) | |
| Did you feel any discrimination *(n=30)* |  |  |
| No | 29(96.7) | |
| Yes | 1(3.3) | |
| Did the clinic have enough facilities *(n=30)* |  |  |
| No | 6(20) | |
| Yes | 24(80) | |
| Did the doctor recall you for follow up *(n=31)* |  |  |
| No | 4(12.9) | |
| Yes | 27(87.1) | |
| Were you satisfied with the services *(n=34)* |  |  |
| Not satisfied |  | 4(11.) |
| Satisfied with care |  | 30(88.2) |
|  | **Mean (SD)** | |
| How much did you understand the information that was given regarding the treatment.  (1=did not understand, 10 Understood) | 8.02 (2.6) | |
| How do you grade do you grade the difficulty in getting to the dental facility/clinic.  (1=very easy, 10 very difficult) | 5.42 (3.3) | |


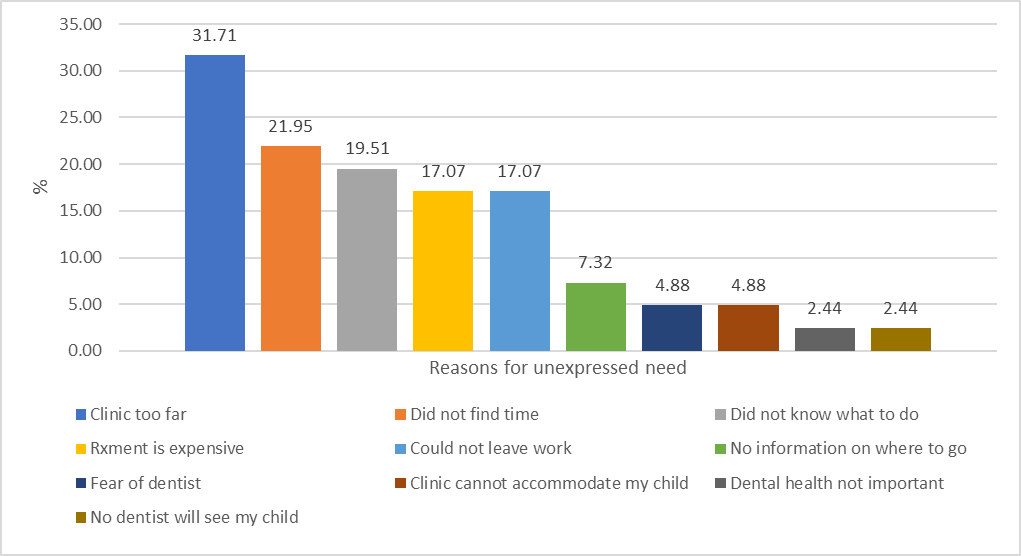


**Fig 1:** Reasons for unexpressed need ***(n 103)***


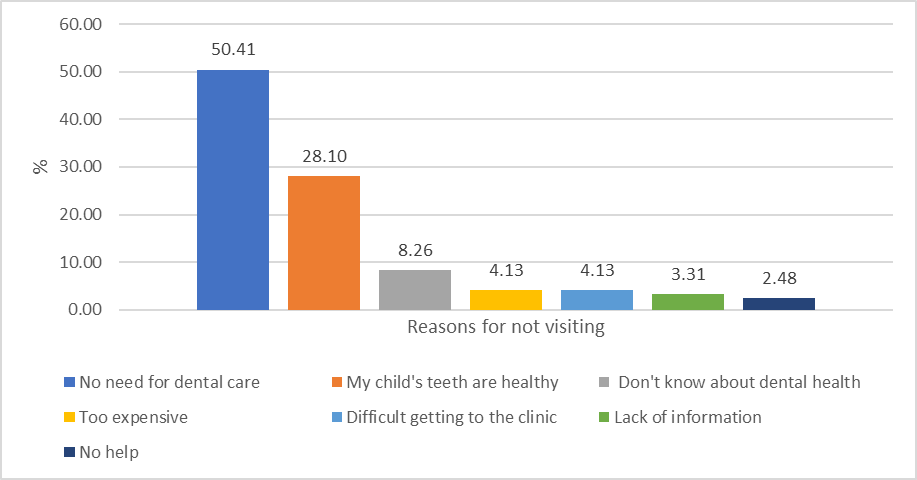


**Fig2:** top seven reasons for not visiting a dental clinic ***(n 121)***.


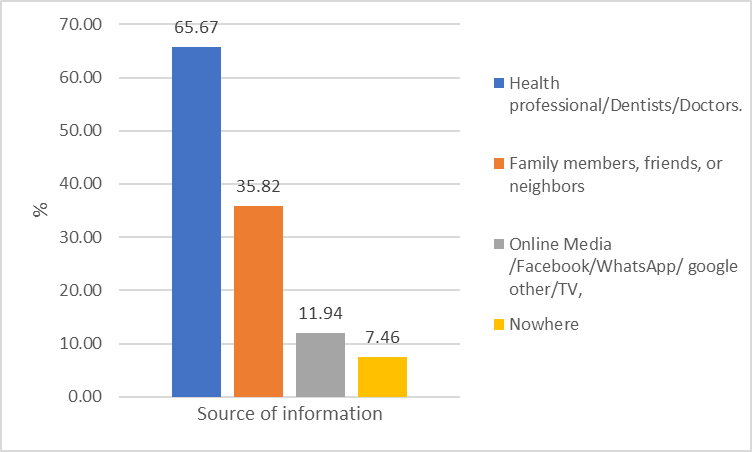


**Fig3:** Source of information regarding dental health ***(n 67)***
